# Do Different Tooth Bleaching–Remineralizing Regimens Affect the Bleaching Effectiveness and Enamel Microhardness *In Vitro*?

**DOI:** 10.1155/2024/6893472

**Published:** 2024-02-14

**Authors:** Hamideh Sadat Mohammadipour, Parnian Shokrollahi, Sima Gholami, Hosein Bagheri, Fatemeh Namdar, Salehe Sekandari

**Affiliations:** ^1^Department of Restorative and Cosmetic Dentistry, School of Dentistry, Mashhad University of Medical Sciences, Mashhad, Iran; ^2^School of Dentistry, Mashhad University of Medical Sciences, Mashhad, Iran; ^3^Dental Materials Research Center, School of Dentistry, Mashhad University of Medical Sciences, Mashhad, Iran; ^4^Dental Research Center, Department of Cosmetic and Restorative Dentistry, School of Dentistry, Mashhad University of Medical Sciences, Mashhad, Iran

## Abstract

**Objective:**

Tooth bleaching may negatively affect the enamel surface properties, such as reduction in hardness values, and remineralizing agents can reverse these effects. This study evaluated the effect of remineralizing agents before, during, and after the bleaching process on enamel's whitening effectiveness and microhardness.

**Methods and Materials:**

The initial color of 104 bovine incisors after immersion in tea solution was recorded, and then, the teeth were randomly divided into eight groups (*n* = 13). Group 1 (NC) was considered the control with no treatment, and Group 2 (B) was bleached with 40% hydrogen peroxide gel. The 3% fluorohydroxyapatite (FHA) and 2% sodium fluoride (NaF) were applied before (FHA/B, NaF/B), during (FHA + B, NaF + B) and after (B/FHA, B/NaF) the bleaching process in other groups. The final color and microhardness in three depths of 20–30, 50–60, and 100–120 *µ*m were measured. Data were analyzed using Shapiro–Wilk, one-way ANOVA, Tukey, Games Howell, repeated measurement, and LSD tests.

**Results:**

The FHA + B presented the lowest *ΔE*, significantly lower than other groups, except B/FHA. The *ΔE* in B/FHA was significantly lower than B/NaF. The bleaching significantly reduced the enamel hardness in three depths. The highest microhardness values were reported for B/NaF and NaF + B, which have no noticeable difference with NC, while FHA/B showed the lowest hardness in three depths, which was significantly lower than NC.

**Conclusion:**

The application of NaF before, during, and after the bleaching improved the microhardness of bleached enamel as the unbleached one with no adverse effect on whitening effectiveness.

## 1. Introduction

The pleasant smile, which is directly affected by the color, shape, and position of teeth [1], may have a crucial role in the self-confidence of patients [2]. Tooth discoloration is one of the main reasons patients seek dental treatment. External, internal, congenital, and acquired factors can adversely destroy the teeth color and the esthetic of the smile [1]. Different treatment modalities have been proposed according to the type and depth of discoloration, from ultraconservative treatments such as scaling, bleaching, and micro and macroabrasions to more invasive treatments such as direct or indirect veneers and all-ceramic crowns [3].

Tooth bleaching is a highly desirable esthetic treatment that can lead to satisfactory results in changing teeth color through the very conservative method using hydrogen peroxide (HP) [4]. HP can diffuse through dental tissues and release active species such as oxygen and per-hydroxyl radicals, causing the oxidation of chromophore molecules and electron-rich unsaturated groups. Splitting the double bonds and rendering the chromophore colorless results in whiter tooth color [5].

There are two bleaching methods, differing in the concentration of the bleaching agent and the period of treatment, including the in-office and the at-home techniques [6]. The at-home bleaching is generally performed with substances that liberate low levels of HP or carbamide peroxide over a long period (generally 2 weeks) and is associated with some advantages such as less concentration of the bleaching substance, lower cost and time, and reduced risk of tooth sensitivity [5]. The in-office bleaching employs high concentrations of bleaching agents (up to 55% HP) and renders immediate and visible results, which increases patient satisfaction with treatment and risk of sensitivity and other potential side effects [5].

Various studies have shown that tooth bleaching agents, especially those with higher HP concentrations and lower pH, can destroy surface morphology, chemical composition, surface hardness, modulus of elasticity, and even fracture toughness of dental tissues [7, 8]. The strong oxidizing effect of HP on the organic matrix of enamel causes enamel changes observed after bleaching. As the enamel's mineral content decreases after bleaching, the resistance of enamel to erosion and surface hardness decreases, and surface roughness increases [9, 10]. These changes make the bleached enamel more susceptible to pigmentation and bacterial adhesion than the unbleached one [11].

Historically, fluoride has been the first attempt in dental practice used for preventive purposes [12]. Recently, casein phosphopeptide-amorphous calcium phosphate [13] and biomimetic hydroxyapatite [14] have been introduced and showed promising results. These compounds have been suggested also in combination of bleaching treatments, in order to reduce side effects. In fact, the use of remineralizing agents, including fluoride [15], CPP-ACP [16, 17], hydroxyapatite [18], and bioactive glass [19], has been recommended before, during, or after the bleaching treatments to reduce these destructive effects. The primary purpose of their application was to improve surface hardness by stimulating remineralization. Besides, bioactive agents containing calcium and phosphorus ions can occlude the dentinal tubules and reduce bleaching-induced sensitivity [20]. It is assumed that using remineralizing materials may reduce the enamel's permeability through the deposition of ionic crystals on the tooth surface, which may limit the penetration of free radicals and, therefore, impair the effectiveness of the bleaching treatment [21]. There are controversies in the literature regarding the effect of remineralizing agents on recovering physical properties and microstructures of enamel and the effectiveness of bleaching [22, 23].

To the best of the authors' knowledge, no information in the literature shows the best remineralizing protocol with the bleaching procedure to have no adverse effect on the bleaching quality and improve enamel hardness. Thus, this *in vitro* study was designed to evaluate the effect of two remineralizing compounds, including sodium fluoride (NaF) and fluorohydroxyapatite (FHA), before, during, and after the bleaching process on tooth color change and microhardness of enamel. The null hypotheses tested in this study are as follows: (1) There was no significant difference between the application of the two mentioned remineralizing agents before, during, and after the in-office bleaching process with 40% HP in aspect of enamel microhardness and (2) these remineralizing–bleaching protocols could not affect the bleaching effectiveness.

## 2. Materials and Methods

### 2.1. Ethical Aspects

This study was conducted at the Department of Cosmetic and Restorative Dentistry (Mashhad University of Medical Sciences, Iran) from 2020 to 2021. The local ethical committee of Mashhad University of Medical Sciences, Iran, independently reviewed and approved this *in vitro* study with the protocol number of IR.MUMS.DENTISTRY.REC.1399.040.

### 2.2. Sample Size Calculation

The outcomes of this study were the amount of tooth color change (*Δ*E) and the microhardness values. Based on previous studies done by Kutuk et al. [24 and Borges et al. [25] who evaluated the effects of in-office bleaching agents combined with different remineralizing agents on enamel, using the *G* ^*∗*^ Power software (latest ver. 3.1.9.7; Heinrich-Heine-Universität Düsseldorf, Düsseldorf, Germany) with an alpha of 0.05, 95% confidence intervals, and 80% power, the minimum sample size in this equivalence study was 13 samples for color evaluation and 11 samples for microhardness assessment in each group. Since both evaluations should be done on the same samples, the 13 samples for each group were allocated.

### 2.3. Sample Preparation

**Figure 1:**
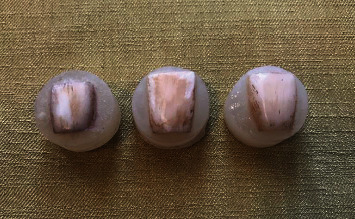
The mounted samples in acrylic resin after immersion in tea solution to simulate the discoloration in this study.

This study was performed on 104 bovine incisors. Before the study initiation, the remaining tissues were removed from dental surfaces. The teeth were evaluated under a stereomicroscope (Dino lite Pro, Anmo Electronic., New Taipei City, Taiwan) with a magnification of 20 to discard those with cracks, enamel defects, caries, etc. The selected teeth were kept in 0.1% thymol solution for 1 week and then immersed in saline solution until the experiment initiation. For microhardness assessment, the roots of the teeth were separated from the crowns, and the crowns were mounted in clear self-cure acrylic resin (Acropars, Marlic Co., Tehran, Iran) so that the labial surface of the tooth was parallel to the horizon. The surfaces were polished using 800; 1,000; and 1,500 grit wet sandpapers (Starcke GmbH and Co. KG, Melle, Germany) under running water. For simulation of discoloration in a laboratory setting, the teeth were placed in a tea solution, which was made by using two tea bags in 50 ml boiling water for 14 consecutive days in an incubator at 37°C (Fine Tech, Shin Saeng., Gyeonggi-do, South Korea) (Figure 1).

### 2.4. Preparation of the In-Office Bleaching Gel

To make 100 ml of the gel containing 40% HP, two separate solutions must first be prepared. For preparing the first solution, 80 ml of 50% HP (Merck., Darmstadt, Germany), 0.068 wt.% Silica (Tamad-Kala Co., Tehran Iran), and 2.3 wt.% Carbopol (Sigmachemical Co., Tehran Iran) were mixed to obtain a uniform solution. Then, glycerin (Merck., Darmstadt, Germany) (thickening agent) was added to the mixture until the volume of the solution reached 98 ml. The mixture was then stirred to increase consistency until a homogeneous and transparent gel was obtained.

For making the second solution, 100 ml of distilled water, 41 wt.% polyethylene glycol (PEG 6000, polyethyleneglycol; Merck., Darmstadt, Germany) and 0.3 M of Tris (tris (hydroxymethyl) aminomethane; Merck., Darmstadt, Germany) were mixed and stirred thoroughly on a magnetic stirrer with a hot plate at 60° C until a uniform solution is obtained. Finally, 2 ml of the second solution was dissolved in the whole of the first solution to raise the pH and form a final gel.

To formulate the activator, 300 Landa manganese oxide (DAE JUN co., Gyeonggi-do, Korea) was dissolved in 2 M Tris with a pH of 10, to which three drops of phenol red (Pars shimi ebtekar gostar., Tehran, Iran) and three drops of methyl orange (Pars shimi ebtekar gostar., Tehran, Iran) were added. Then, 10% weight of CMC (Tamad-Kala Co., Tehran Iran) was added to it, and finally, the above mixture was mixed by a magnetic stirrer on a hot plate at a temperature of 60 degrees until its texture was completely jellylike.

### 2.5. The Tooth Bleaching–Remineralizing Protocols

The discolored and mounted teeth were randomly divided into eight groups (*n* = 13). Randomization was done using the random number table. It should be mentioned that all pastes containing remineralizing agents were formulated and used by the study researchers. The paste containing 2% sodium fluoride (NaF) was formulated by mixing NaF (Merck, Darmstadt, Germany) with CMC (Tamad-Kala Co., Tehran Iran) as thickener. Fluorohydroxyapatite (FHA) synthesized using a sol–gel method based on a study done by Shekofteh et al. [26], and the remineralizing paste containing 3% fluorohydroxyapatite (FHA) was formulated by mixing FHA (Merck, Darmstadt, Germany) with CMC (Tamad-Kala Co., Tehran Iran) as thickener. The bleaching protocols with the remineralizing materials were performed in different ways as follows:

  Group 1 (NC) was considered as the control with no treatment (negative control).
  Group 2 (B): In this group, the formulated bleaching gel containing 40% HP was used for 45 min (positive control).
  Group 3 (FHA/B): In the third group, before applying the bleaching gel, the remineralizing paste containing 3% fluorohydroxyapatite (FHA) was used for 30 min. Then, the bleaching treatment was done the same as the B group.
  Group 4 (FHA + B): In this group, a physical mixture of the bleaching gel and 3% FHA was used for 45 min.
  Group 5 (B/FHA): The paste containing 3% FHA was applied for 30 min after the bleaching procedure with application of 40% HP.
  Group 6 (NaF/B): The paste containing 2% sodium fluoride (NaF) was applied on the surface of the samples before applying the bleaching gel containing 40% HP.
  Group 7 (NaF + B): In this group, a physical mixture of the bleaching gel and 2% NaF was used for 45 min.
  Group 8 (B/NaF): The paste containing 2% NaF was applied to the bleached samples for 30 min after applying the bleaching gel.

**Figure 2:**
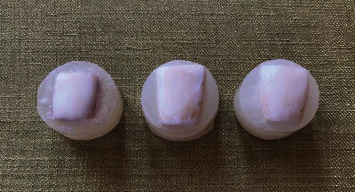
The mounted samples in acrylic resin after the different bleaching–remineralizing protocols.

During the bleaching process in the experimental groups, the bleaching gel was stirred on the surface of the teeth every 15 min to refresh the gel in contact with the enamel surface. At the end of the various bleaching–remineralizing regimens, the gel remnants were removed from the enamel surface, and the teeth were thoroughly rinsed with water (Figure 2).

### 2.6. Color Evaluation

In this study, the tooth color change was measured by an instrumental method using a colorimeter (Chroma meter, KONICA MINOLT., Hino-shi Tokyo, Japan) and a standard white background to place under the specimens. Impressions of the specimens were taken with high-putty silicon (Speedex, Coltene., Alstatten, Switzerland) to standardize the evaluation. A window was created on the labial surface of the silicon guide corresponding to the middle third of the incisors using a metal device with well-formed borders and 11 mm in diameter, which has a similar diameter to the colorimeter tip. This window accommodated the tip of the colorimeter for a standard color evaluation [5, 27].

*L* ^*∗*^, *a* ^*∗*^, and *b* ^*∗*^ values of the CIE Lab system were recorded for each tooth. *L* ^*∗*^ indicates the brightness, and *a* ^*∗*^ and *b* ^*∗*^ represent hue. *a* ^*∗*^ axis represents saturation in the red-green axis, and *b* ^*∗*^ is the saturation in the blue-yellow axis. The color variables of each specimen were recorded after being immersed in the tea solution (T1) and after performing various remineralizing–bleaching treatments (T2). The difference of *L* ^*∗*^, *a* ^*∗*^, and *b* ^*∗*^ between the T1 and T2 expressed as *ΔL*, *Δa*, and *Δb*, respectively (*ΔL* = *L* final − *L* initial; *Δa* = *a* final − *a* initial; *Δb* = *b* final − *b* initial). The color difference (*ΔE*) was calculated using the following equation:

(1) $$\begin{array}{c} \Delta E={\left[{\left(\Delta L\right)}^{2}+{\left(\Delta a\right)}^{2}+{\left(\Delta b\right)}^{2}\right]}^{\frac{1}{2}} \end{array}$$

Greater *ΔE* values show better whitening effectiveness evidenced by increasing *L* ^*∗*^ value and decreasing *a* ^*∗*^ and *b* ^*∗*^ values.

### 2.7. Microhardness Evaluation

**Figure 3:**
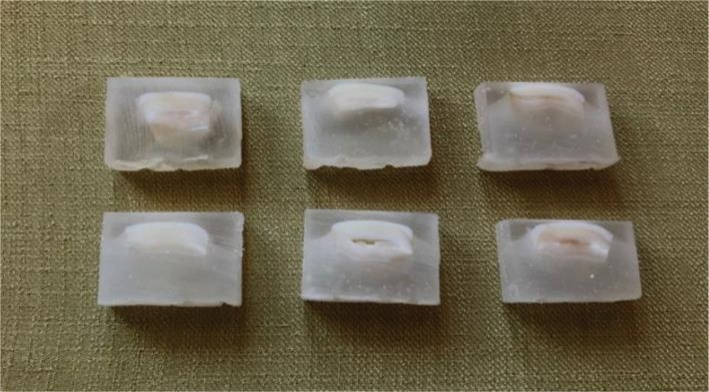
The embedded and cut samples in clear acrylic resin which were ready for the microhardness evaluations in three different depth of enamel.

To perform the microhardness test, the enamel surface of each sample was coated with an acrylic resin, and a vertical cut was made in each sample. This surface was then smoothed by 800; 1,000; 1,500; and 2,000 grit sandpapers (Starcke GmbH and Co. KG, Melle, Germany) and then polished with a felt disk and polishing paste to obtain a perfectly polished surface (Figure 3). Vickers microhardness values were obtained by hardness tester device (microhardness tester, KOOPA PAZHOOHESH., Tehran, Iran, Model: MH3). The Vickers hardness number (VHN) was determined by fitting a 10 N force into the diamond indenter and by allowing the indenter to rest on the enamel surface for 10 s at three different depths of 20–30 (D1), 50–60 (D2), and 100–120 *µ*m (D3) [28]. To increase the accuracy of the results, the microhardness test was repeated three times in each sample, and their average was recorded.

### 2.8. Statistical Analysis

The Shapiro–Wilk statistical test was used to evaluate the normal distribution of data. To compare the color changes between different experimental groups, one-way analysis of variance (ANOVA), Tukey, and Games Howell tests were used. To compare the microhardness changes in different depths of enamel, repeated measurement and LSD analysis were used at a significant level of 0.05. The data were analyzed through SPPS software (version 16.0; SPSS Inc., Chicago, IL, USA).

## 3. Results

Based on Shapiro–Wilk analysis results, the normal distribution of data regarding the color change and hardness in all study groups was confirmed (*P*  > 0.05).

### 3.1. The Bleaching Effectiveness

The mean, standard deviation, minimum, and maximum of total color change (*ΔE*) in all study groups were presented in Table 1. Based on one-way analysis of variance, there was a significant difference between the study groups (*P*  < 0.001). As shown, the highest and lowest mean *ΔE* was recorded in B/NaF (10.19 ± 3.34) and FHA + B (4.91 ± 1.34) groups, respectively. Pairwise comparisons of mean *ΔE* values by Tukey HSD test revealed that *ΔE* in group B was significantly higher than the FHA + B group (*P*  < 0.001) but was not significantly different from other groups. Also, the amount of *ΔE* in the FHA + B group was significantly lower than in the three groups treated with NaF (*P*  < 0.01) and also the FHA/B group (*P*=0.040). The mean *ΔE* value in the B/FHA group was significantly lower than the B/NaF group (*P*=0.035).

The mean, standard deviation, minimum, and maximum of color variables (*ΔL*, *Δa*, *Δb*) in all study groups were presented in Table 2. Based on one-way analysis of variance, there was a significant difference between the study groups (*P*  < 0.001). Pairwise comparisons of mean *ΔL* values by Tukey HSD test revealed that *ΔL* in group B was significantly higher than the FHA + B group (*P*=0.001) but was not significantly different from other groups (*P*  > 0.05). Also, the amount of *ΔL* in the FHA + B group was significantly lower than all experimental groups (*P*  < 0.05) except B/FHA group (*P*=0.153). *Δa* in group B was significantly higher than the FHA/B group (*P*  < 0.001) but was not significantly different from other groups (*P*  > 0.05). The amount of *Δa* in the FHA/B group was significantly lower than all experimental groups (*P*  < 0.05). *Δb* in group B was significantly higher than the FHA + B group (*P*  < 0.001) and lower than NaF + B group (*P*=0.001) but was not significantly different from other groups (*P*  > 0.05). Also, the amount of *Δb* in the FHA + B group was significantly lower than all experimental groups (*P*  < 0.05) except FHA/B group (*P*=0.993). The amount of *Δb* in the NaF + B group was significantly higher than all experimental groups (*P*  < 0.05).

### 3.2. The Microhardness Changes

The mean and standard deviation of the microhardness values in different study groups and depths are presented in Table 3. The mean of the microhardness value in each group was significantly different between the three measurement depths (*P* < 0.001). In each depth of measurement, there was a significant difference between the study groups (*P* < 0.001). Also, the interaction between the group and the depth of assessment was significant. Based on the obtained results from the LSD test in D1 (20–30 *µ*m), the NC group's mean microhardness was significantly higher than all the study groups except NaF + B and B/NaF groups. The mean microhardness in group FHA/B was lower than B/FHA, FHA + B, NaF/B, and B/NaF groups. In D2 (50–60 *µ*m), the mean microhardness in the FHA/B group was significantly lower than all groups, and in group B, it was significantly lower than all groups except the FHA/B group. In D3 (100–120 *µ*m), the mean microhardness in the FHA/B group was significantly lower than other groups except group B, and the mean microhardness in group B was significantly lower than NC, B/NaF, and B/FHA groups. No significant differences were observed in other pairwise comparisons and depths. The pairwise comparison of mean microhardness between different depths showed that the average value in superficial depth was significantly lower in all groups than in the deeper depth.

## 4. Discussion

This *in vitro* study aimed to address the concerns regarding the negative effect of the remineralizing agents when used before, after, or during the bleaching treatment, which may interfere with the penetration of the bleaching materials into the dental tissues and reduce the bleaching effectiveness. The obtained results showed that the application of two tested remineralizing agents including 2% NaF and 3% FHA before, during, and after the in-office bleaching with 40% HP improved the microhardness of bleached enamel, except when 3% FHA was applied before the bleaching process. Thus, the first null hypothesis of this study was rejected. Since the remineralizing protocols showed no detrimental effect on the bleaching effectiveness, except in the group where FHA was used during bleaching, the second null hypothesis was also partially rejected.

In the current research, bleaching effectiveness was measured using an instrumental method of color evaluation through colorimetery. This method is accurate, reproducible, and objective compared with the visual method, providing standardization of shade analysis by precise measurements in CIE *L*^*∗*^*a*^*∗*^*b*^*∗*^ units. Indeed, the visual color selection was negatively affected by some factors, including gender, age, eye fatigue, ambient light, angle of lighting, and lack of reliable shade guides, while instruments such as spectrophotometers and colorimeters can exclude these errors, and thus, unbiased and reproducible results can be achieved [29].

The average total color change (*ΔE*) of experimental groups in the current research was 4.91 ± 1.34–10.19 ± 3.34. Since the *ΔE* greater than 3.3 or 3.7 produces clinically perceptible color changes in humans [29], the whole experimental bleaching–remineralizing regimens revealed noticeable color changes after the bleaching process, which were higher than the human eye threshold, and it confirmed the bleaching effectiveness of the tested protocols. Greater *ΔE* values show better whitening effectiveness evidenced by increasing *L* value. Positive *ΔL* means the specimens became whiter, while negative *ΔL* means specimens became darker. The highest *ΔE* and *ΔL* values were recorded for three NaF-treated groups, which showed no significant difference with the bleached control group (B group). In contrast, the least amount of *ΔE* and *ΔL* was observed in the FHA + B group, which was significantly lower than the other groups except the B/FHA group. The low color change in FHA + B may be attributed to the adverse interactions between HP and this material. In addition, it seems the application of FHA may induce some degree of discoloration when used immediately after the bleaching treatment (B/FHA group). A previous study showed the utilizing of casein phosphopeptide-amorphous calcium phosphate fluoride (CPP-ACPF) paste, Remin Pro paste (containing hydroxylapatite, fluoride, and xylitol), and 0.05% NaF mouthwash caused noticeable teeth discoloration immediately after bleaching [30]. This outcome somewhat contrasted with several previous studies, which showed the lack of adverse effects of fluoride before or during the bleaching treatment on the bleaching effectiveness [8, 11, 21, 31]. For instance, Chen et al. [21] indicated that using fluoride during and after the bleaching with 10% carbamide peroxide has no significant difference in bleaching efficacy. The same findings have been reported when other remineralizing agents were used [18, 24, 29, 32]. Ferraz et al. [18] showed that adding nano-hydroxyapatite (N-HA) to the bleaching gel containing 35% HP had no detrimental effect on the tooth bleaching rate. The same results have been reported by Vano et al. [33] when 2% N-HA was added to the bleaching gel containing 6% HP. In agreement, the combination of NaF, CPP-ACPF, and N-HA with the bleaching gels did not affect the bleaching effectiveness in the Missili et al. [31] study. In line, other studies revealed no significant difference in bleaching efficacy when using N-HA before or during bleaching with HP [18, 30, 31, 33].

Despite significantly lower *ΔE* values in FHA + B than B as the positive control group, the lack of significant difference between B and other study groups confirmed that the remineralizing treatments had no adverse effect on the bleaching effectiveness of the bleaching gels. Therefore, it can be concluded that applying 2% NaF before, during, and after the bleaching procedure and applying 3% FHA before the bleaching treatment did not have a detrimental effect on whitening effectiveness.

Microhardness, as a suitable and standard test, is used to assess the minor alterations in enamel, such as initial demineralization, especially for evaluating the effect of remineralizing agents in bleaching treatments. The free radicals released by HP gels act not only over the chromophores but also over the organic structure of enamel, oxidizing the proteins, which resulted in structural changes in the protein matrix located between the enamel crystallites, interfering with the crystal structure and consequently making it more susceptible to mechanical impacts such as reductions in microhardness and fracture toughness [34]. Based on ISO 28399 [34], which refers to bleaching procedures, the acceptable reduction of microhardness promoted by bleaching gels shall not be higher than 10%. In contrast, in the present study, microhardness reduction, especially in D1 in bleached enamel, was remarkably lower than in unbleached samples. Despite most of the previous studies that evaluated the effect of remineralizing agents in superficial bleached enamel, this study assessed microhardness in three depths of enamel to show the penetration depth of the remineralizing materials into the enamel. The obtained results of hardness evaluation showed the lowest mean value of microhardness in D1, which was significantly increased in D2 and D3. It was attributed to the fact that the highest concentration of the bleaching and oxidizing agents concentrated on the tooth's surface, and from the surface to the depth of enamel, their concentrations decreased. During the bleaching process, oxidizing molecules penetrate the dental hard tissues through interprismatic spaces and subsurface pores of the enamel and even reach the pulp cells, which cause reversible pulpitis and consequent sensitivity. The sealing of exposed dentin or enlarged dentinal tubules with remineralizing agents prohibited the peroxide molecules' diffusion pathways and reduced the incidence of sensitivity [35]. The reduction in peroxide diffusion and enamel microhardness was confirmed by Torres et al. [34] when the bleaching gel containing calcium and fluoride was investigated. Irmaleny et al. [36] have observed that there was an increase in the enamel hardness when bleached enamel is remineralized with CPP-ACPF and 5% sodium fluoride (NaF). Melo et al. [37] found that application of remineralizing products (Tooth Mousse, Remin-Pro, Colgate Pro-Relif, Mirafluor) generates a significant increase in enamel microhardness. Also Yang et al. [38] suggested that hydrated calcium silicate could minimize the microhardness loss of tooth structure caused by bleaching agents.

Conversely, Furlan et al. [22] showed that bleaching gels containing fluoride or calcium did not prevent the reduction of the surface hardness induced by bleaching. In D2 and D3 measurements, the microhardness of the FHA/B group was significantly lower than other groups and remained at the level of the bleached enamel. This was in contrast with Crastechini et al. [8] and Gomes et al. [39] study, which showed that the application of fluoride and hydroxyapatite before the bleaching improved the enamel hardness compared to the control group. However, the enamel alterations resulting from the bleaching treatment should be considered, such as a decline in enamel hardness confined to the superficial layer of enamel and a return to the situation before the bleaching treatment in a short time, even during 2 weeks [24]. Although saliva, which contains calcium, fluoride, and other remineralizing agents, may reduce the negative effect of the bleaching agents on the superficial layer of enamel, the application of the remineralizing agents can prevent hardness reduction or even improve it to the level of unbleached enamel in shorter time. On the other hand, applying these remineralizing agents may reduce tooth bleaching-induced sensitivity by occluding open dentinal tubules and sealing the surface. However, due to the laboratory design of this study, it could not assess in this study and needs further research. Since, in this study, the hardness changes in different depth were evaluated by cutting the tooth samples, the time for returning the hardness of bleached enamel to the normal values presented in previous studies could not be investigated, and it should be considered as a limitation of this study.

One of the most critical limitations of this study was immediate evaluations of tooth color change after the bleaching–remineralizing protocols. The bleaching with a high concentration of HP resulted in some degrees of dehydration of teeth, so the color assessment immediately after the bleaching termination may show brighter colors. The results of this study confirmed the positive effect of two remineralizing agents on reversing the reduced bleached enamel microhardness. Since remineralizing agents can positively affect reducing sensitivity, their effect on reducing bleaching-induced sensitivity should be considered in further clinical trials. Furthermore, comparing these two agents with other remineralizing materials in terms of surface enamel properties, such as hardness and bleaching effectiveness, may be investigated in future research.

## 5. Conclusion

Based on the findings of this study and considering the limitations of *in vitro* studies:

1. The use of remineralizing agents such as 2% sodium fluoride and 3% fluorohydroxyapatite before, during, and after the in-office bleaching with 40% hydrogen peroxide had no detrimental effect on the bleaching effectiveness, except in the group in which the bleaching gel containing fluorohydroxyapatite was used.
2. Both remineralizing agents improved the microhardness of bleached enamel, except when 3% fluorohydroxyapatite was applied before the bleaching process.
3. Applying 2% sodium fluoride before, after, or combined with the bleaching treatment should be advocated to effectively enhance bleached enamel microhardness up to unbleached ones and produce the same bleaching effectiveness as the nonremineralized bleached enamel.

## Figures and Tables

**Table 1:** Means, standard deviations (SD), minimum and maximum values of total color changes (*ΔE*) among the experimental groups.

Study groups	Number	Mean ± SD	Min	Max	One-way analysis of variance (ANOVA)
B	13	9.64 ± 2.55^ab^*∗*^^	5.49	13.59	*F* = 6.68, *P* < 0.001
FHA/B	13	7.90 ± 2.48^ab^	4.74	12.07	
FHA + B	13	4.91 ± 1.34^c^	2.30	6.74	
B/FHA	13	7.16 ± 1.73^cb^	4.42	9.82	
NaF/B	13	8.34 ± 2.63^ab^	4.54	12.81	
NaF + B	13	9.21 ± 2.58^ab^	4.94	14.75	
B/NaF	13	10.19 ± 3.34^a^	4.25	16.09	

^*∗*^Mean values with the same superscript letters are not statistically significant at *P* > 0.05.

**Table 2:** Means, standard deviations (SD), minimum and maximum values of color variables (*ΔL*, *Δa*, *Δb*) among the experimental groups.

Study groups	Mean *ΔL* ± SD	Mean *Δa* ± SD	Mean *Δb* ± SD
B	8.90 ± 2.59^a^*∗*^^	−2.15 ± 0.71^ac^	−2.80 ± 0.66^a^
FHA/B	7.56 ± 2.45^a^	−0.71 ± 0.47^b^	−1.60 ± 1.46^ab^
FHA + B	4.42 ± 1.42^b^	−1.50 ± 0.75^a^	−1.27 ± 0.51^b^
B/FHA	6.23 ± 1.8^ab^	−1.73 ± 0.55^a^	−2.82 ± 0.80^a^
NaF/B	7.55 ± 2.69^a^	−1.56 ± 0.75^a^	−2.90 ± 0.89^a^
NaF + B	7.65 ± 2.73^a^	−2.58 ± 0.69^c^	−4.21 ± 0.79^c^
B/NaF	9.50 ± 3.46^a^	−1.76 ± 0.73^ac^	−2.88 ± 0.93^a^
One-way analysis of variance (ANOVA)	*F* = 21.43, *P* < 0.001	*F* = 24.53, *P* < 0.001	*F* = 29.92, *P* < 0.001

^*∗*^Mean values with the same superscript letters are not statistically significant at *P* > 0.05.

**Table 3:** The means and standard deviations of microhardness values among the experimental groups at three different depths of measurements.

Study groups	D1 (20–30 *µ*m)	D2 (50–60 *µ*m)	D3 (120–100 *µ*m)	Repeated measurement analysis
NC	327.4 ± 52.0^aA^	336.7 ± 49.4^aB^	399.7 ± 44.1^aC^	*F* = 60.8, *P* < 0.001
B	174.3 ± 35.9^bA^	273.8 ± 24.0^bB^	336.5 ± 13.9^bcC^	*F* = 175.34, *P* < 0.001
FHA/B	230.9 ± 11.8^cA^	284.0 ± 15.4^bB^	324.9 ± 20.9^cC^	*F* = 226.97, *P* < 0.001
FHA + B	256.5 ± 18.3^dA^	329.1 ± 15.4^aB^	356.4 ± 20.0^abC^	*F* = 148.06, *P* < 0.001
B/FHA	253.7 ± 17.2^dA^	338.2 ± 24.0^aB^	359.0 ± 14.7^aC^	*F* = 161.52, *P* < 0.001
NaF/B	268.4 ± 33.6^dA^	342.5 ± 30.1^aB^	362.0 ± 30.3^abC^	*F* = 210.57, *P* < 0.001
NaF + B	271.7 ± 48.8^acdA^	345.4 ± 39.1^aB^	364.6 ± 34.4^abC^	*F* = 156.14, *P* < 0.001
B/NaF	283.4 ± 41.1^adA^	348.1 ± 22.8^aB^	362.8 ± 16.9^aC^	*F* = 87.83, *P* < 0.001
One-way analysis of variance	*F* = 20.17, *P* < 0.001	*F* = 15.65, *P* < 0.001	*F* = 9.02, *P* < 0.001	Interaction *F* = 11.55, *P* < 0.001

^*∗*^The different lowercase superscript letters in the columns indicate statistically significant differences at *P*  < 0.05.  ^*∗*^The different uppercase superscript letters in the rows indicate statistically significant differences at *P*  < 0.05.

## Data Availability

The data used to support the findings of this study are available from the corresponding author upon request.

## References

[1] Polydorou O., Monting J. S., Hellwig E., Auschill T. M. (2007). Effect of in-office tooth bleaching on the microhardness of six dental esthetic restorative materials. *Dental Materials Journal*.

[2] Favaro J. C., Geha O., Guiraldo R. D., Lopes M. B., Aranha A. M. F., Berger S. B. (2019). Evaluation of the effects of whitening mouth rinses combined with conventional tooth bleaching treatments. *Restorative Dentistry & Endodontics*.

[3] Hughes F. J. (2005). Color atlas of dental medicine: periodontology. *British Dental Journal*.

[4] Ade Araújo L. S. N., dos Santos P. H., Anchieta R. B. (2013). Mineral loss and color change of enamel after bleaching and staining solutions combination. *Journal of Biomedical Optics*.

[5] Ahrari F., Akbari M., Mohammadipour H. S., Fallahrastegar A., Sekandari S. (2020). The efficacy and complications of several bleaching techniques in patients after fixed orthodontic therapy. A randomized clinical trial. *Swiss Dental Journal*.

[6] Attin T., Hannig C., Wiegand A., Attin R. (2004). Effect of bleaching on restorative materials and restorations—a systematic review. *Dental Materials Journal*.

[7] Heshmat H., Ganjkar M. H., Miri Y., Fard M. J. K. (2016). The effect of two remineralizing agents and natural saliva on bleached enamel hardness. *Dental Research Journal*.

[8] Crastechini E., Borges A. B., Torres C. R. G. (2019). Effect of remineralizing gels on microhardness, color and wear susceptibility of bleached enamel. *Operative Dentistry*.

[9] Smidt A., Feuerstein O., Topel M. (2011). Mechanical, morphologic, and chemical effects of carbamide peroxide bleaching agents on human enamel in situ. *Quintessence International*.

[10] Ferreira S. S., Araujo J. L., Morhy O. N., Tapety C. M. C., Youssef M. N., Sobral M. A. P. (2011). The effect of fluoride therapies on the morphology of bleached human dental enamel. *Microscopy Research and Technique*.

[11] Berger S. B., Coelho A. S., Oliveira V. A., Cavalli V., Giannini M. (2008). Enamel susceptibility to red wine staining after 35% hydrogen peroxide bleaching. *Journal of Applied Oral Science*.

[12] Zampetti P., Scribante A. (2020). Historical and bibliometric notes on the use of fluoride in caries prevention. *European Journal of Paediatric Dentistry*.

[13] Fallahzadeh F., Heidari S., Najafi F., Hajihasani M., Noshiri N., Nazari N. F. (2022). Efficacy of a novel bioactive glass–polymer composite for enamel remineralization following erosive challenge. *International Journal of Dentistry*.

[14] Butera A., Pascadopoli M., Pellegrini M. (2022). Biomimetic hydroxyapatite paste for molar–incisor hypomineralization: a randomized clinical trial. *Oral Diseases*.

[15] Kyaw K. Y., Otsuki M., Segarra M. S., Tagami J. (2018). Effect of sodium fluoride pretreatment on the efficacy of an in-office bleaching agent: an in vitro study. *Clinical and Experimental Dental Research*.

[16] Kaur G., Sanap A. U., Aggarwal S. D., Kumar T. (2015). Comparative evaluation of two different remineralizing agents on the microhardness of bleached enamel surface: results of an in vitro study. *Indian Journal of Dental Research*.

[17] Cunha A. G. G., De Vasconcelos A. A. M., Borges B. C. D. (2012). Efficacy of in-office bleaching techniques combined with the application of a casein phosphopeptide-amorphous calcium phosphate paste at different moments and its influence on enamel surface properties. *Microscopy Research and Technique*.

[18] Ferraz L. N., Júnior W. F. V., Ambrosano G. M. B., Giorgi M. C. C., Aguiar F. H. B., Lima D. A. N. L. (2018). Effect of different concentrations of nanohydroxyapatite on tooth bleaching effectiveness and enamel bond strength. *Brazilian Dental Science*.

[19] Rastelli A. N. S., Nicolodelli G., Romano R. A. (2016). After bleaching enamel remineralization using a bioactive glass–ceramic (BioSilicate®). *Biomedical Glasses*.

[20] Alexandrino L. D., Alencar C. M., Silveira A., Alves E. B., Silva C. M. (2017). Randomized clinical trial of the effect of NovaMin and CPP-ACPF in combination with dental bleaching. *Journal of Applied Oral Science*.

[21] Chen H.-P., Chang C.-H., Liu J.-K., Chuang S.-F., Yang J.-Y. (2008). Effect of fluoride containing bleaching agents on enamel surface properties. *Journal of Dentistry*.

[22] Furlan I. S., Bridi E. C., Amaral F., Franca F. M. G., Turssi C. P., Basting R. T. (2017). Effect of high- or low-concentration bleaching agents containing calcium and/or fluoride on enamel microhardness. *General Dentistry*.

[23] Vieira I., Vieira-Junior W., Pauli M. (2020). Effect of in-office bleaching gels with calcium or fluoride on color, roughness, and enamel microhardness. *Journal of Clinical and Experimental Dentistry*.

[24] Kutuk Z. B., Ergin E., Cakir F. Y., Gurgan S. (2018). Effects of in-office bleaching agent combined with different desensitizing agents on enamel. *Journal of Applied Oral Science*.

[25] Borges B. C. D., Borges J. S., de Melo C. D. (2011). Efficacy of a novel at-home bleaching technique with carbamide peroxides modified by CPP-ACP and its effect on the microhardness of bleached enamel. *Operative Dentistry*.

[26] Shekofteh K., Boruziniat A., Moghaddas M.-J., Namdar F., Zahabi E., Bagheri H. (2018). Formulation and mechanical characterization of a semi-crystalline nano-fluorine hydroxyapatite-filled dental adhesive. *Journal of the Australian Ceramic Society*.

[27] Da Cunha F. B., Rodrigues e Silva B. H., De Paula B. L. F., Alencar C. M., de Albuquerque Jassé F. F., Silva C. M. (2018). Effect of high concentrated fluoride-based dentifrice on the hardness, roughness, and color of the bleached enamel. *Journal of Conservative Dentistry*.

[28] Delfino C. S., Chinelatti M. A., Carrasco-Guerisoli L. D., Batista A. R., Fröner I. C., Palma-Dibb R. G. (2009). Effectiveness of home bleaching agents in discolored teeth and influence on enamel microhardness. *Journal of Applied Oral Science*.

[29] Pruthi G., Jain V., Kandpal H. C., Mathur V. P., Shah N. (2010). Effect of bleaching on color change and surface topography of composite restorations. *International Journal of Dentistry*.

[30] Malekipour M., Norouzi Z., Shahlaei S. (2019). Effect of remineralizing agents on tooth color after home bleaching. *Frontiers in Dentistry*.

[31] Misilli T., Çarıkçıoğlu B., Deniz Y., Aktaş Ç. (2022). The impact of remineralization agents on dental bleaching efficacy and mineral loss in bleached enamel. *European Journal of Oral Sciences*.

[32] Sasaki R. T., Catelan A., Bertoldo E. S. (2015). Effect of 7.5% hydrogen peroxide containing remineralizing agents on hardness, color change, roughness and micromorphology of human enamel. *American Journal of Dentistry*.

[33] Vano M., Derchi G., Barone A., Genovesi A., Covani U. (2015). Tooth bleaching with hydrogen peroxide and nano-hydroxyapatite: a 9-month follow-up randomized clinical trial. *International Journal of Dental Hygiene*.

[34] Torres C., Zanatta R. F., Silva T. J., Borges A. B. (2019). Effect of calcium and fluoride addition to hydrogen peroxide bleaching gel on tooth diffusion, color, and microhardness. *Operative Dentistry*.

[35] Mohammadipour H. S., Bagheri H., Khorshid M., Akbari M., Akhlaghi S., Khammar M. S. (2023). Tooth sensitivity and whitening effect of an in-office bleaching gel containing sodium hexametaphosphate: a randomized triple-blind clinical trial. *Journal of Dental Materials and Techniques*.

[36] Irmaleny I., Hidayat O. T., Yolanda Y., Tobing E. L. (2023). Comparative evaluation of the increase in enamel hardness post-external bleaching after using casein phosphopeptide amorphous calcium phosphate fluoride (CPP-ACPF) and 5% sodium fluoride (NaF) remineralizing agents. *European Journal of Dentistry*.

[37] Melo M., Fioresta R., Sanz J. L., Pecci-Lloret M. P., Llena C. (2022). Effect of highly concentrated bleaching gels on enamel microhardness and superficial morphology, and the recovery action of four remineralizing agents. *BMC Oral Health*.

[38] Yang S.-Y., Choi J.-W., Kim K.-M., Kwon J.-S. (2022). Effects of 35% hydrogen peroxide solution containing hydrated calcium silicate on enamel surface. *Clinical Oral Investigations*.

[39] Gomes Y. S. L., Alexandrino L. D., Alencar C. M., Alves E. B., Faial K. C., Silva C. M. (2017). In situ effect of nanohydroxyapatite paste in enamel teeth bleaching. *The Journal of Contemporary Dental Practice*.

